# Conversion of glutamate into proline by the leucine analog BCH enhances biphasic insulin secretion in pancreatic β-cells

**DOI:** 10.1016/j.jbc.2025.108449

**Published:** 2025-03-26

**Authors:** Sevda Gheibi, Luis Rodrigo Cataldo, Hamidreza Ardalani, Lisa Nocquet, Peter Spégel, Susanne G. Straub, Geoffrey W.G. Sharp, Malin Fex, Hindrik Mulder

**Affiliations:** 1 Unit of Molecular Metabolism, Lund University Diabetes Centre, Malmö, Sweden; 2 Novo Nordisk Foundation Center for Basic Metabolic Research, Faculty of Health and Medical Sciences, University of Copenhagen, Copenhagen, Denmark; 3 Department of Chemistry, Centre for Analysis and Synthesis, Lund University, Lund, Sweden; 4 Pharmacokinetics, Dynamics, and Metabolism, Pfizer R&D, Pfizer Inc., Bothell, Washington, USA; 5 Department of Molecular Medicine, Cornell University, Ithaca, New York, USA

**Keywords:** β-cells, BCH, glutamate dehydrogenase, proline, insulin secretion

## Abstract

Biphasic insulin secretion, which fails in type 2 diabetes, can be provoked by various nutrient stimuli, glucose being the superior physiological one. To identify pathways that may play a role in **β**-cell stimulus-secretion coupling, we compared **β**-cell and islet functional, secretory, and metabolic responses to glucose and 2-aminobicyclo-(2,2,1)-heptane-2-carboxylic acid (BCH), a leucine analog, that acts as an allosteric activator of glutamate dehydrogenase. We employed a range of techniques, including insulin secretion assays, mitochondrial activity measurements, ATP/ADP ratio assessments, and cytosolic Ca^2+^ level quantifications. Metabolomics was used to analyze cellular metabolite profiles in response to glucose and BCH. Additionally, we investigated the role of proline synthesis by silencing *ALDH18A1*, encoding proline 5-carboxylate synthase, in both clonal **β**-cells and human islets. BCH and glucose similarly induced a biphasic insulin response in INS-1 832/13 cells, paralleled by increased mitochondrial activity and raised ATP/ADP ratios, plasma membrane depolarization, and elevated cytosolic Ca^2+^ levels. Metabolomics revealed that proline levels increased significantly only in BCH-stimulated **β-**cells. Silencing *ALDH18A1* disrupted insulin secretion in response to both glucose and BCH, accompanied by reduced cytosolic Ca^2+^ levels, ATP/ADP ratios, and mitochondrial activity. Our findings demonstrated that BCH-induced activation of glutamate dehydrogenase leads to the conversion of glutamate into proline, which apparently enhances **β**-cell stimulus-secretion coupling. This work identifies a previously unrecognized role of proline metabolism in **β**-cell function and provides novel insights into the complex regulation of insulin secretion.

Diabetes is a critical global health issue, with recent calculations indicating that 828 million people individuals were living with the disease in 2022 (1). Central to the pathogenesis of diabetes is the dysfunction of pancreatic β-cells, which are crucial for regulating blood glucose levels through insulin secretion. A key feature of β-cell dysfunction in diabetes is the impairment of biphasic insulin secretion, which consists of an initial rapid release followed by a sustained phase. This biphasic pattern is essential for maintaining blood glucose homeostasis, and its failure is linked to the onset and progression of type 2 diabetes (2, 3). Despite advances in the understanding of β-cell function, the precise regulatory mechanisms, particularly the involvement of different metabolic pathways in insulin secretion, remain incompletely understood (2, 3, 4, 5).

Insulin secretion in β-cells is primarily stimulated by glucose, which initiates a cascade of metabolic events. The first rapid phase of insulin release is triggered by an immediate increase in glucose metabolism, leading to the generation of ATP through glycolysis and oxidative phosphorylation. This increase in ATP results in the closure of ATP-sensitive potassium channels (K_ATP_), causing membrane depolarization, an influx of Ca^2+^ into the cytosol, and exocytosis of insulin (6, 7). The second, sustained phase involves additional metabolic pathways that amplify the initial response. One of the key metabolites implicated in this amplifying phase is glutamate (8, 9).

Glutamate plays a dual role in β-cells: it serves as a precursor for α-ketoglutarate, a critical component of the tricarboxylic acid (TCA) cycle, and as a signaling molecule that influences insulin secretion. The enzyme glutamate dehydrogenase (GDH) catalyzes the conversion of glutamate to α-ketoglutarate, linking glutamate metabolism to energy production and stimulus-secretion coupling (10, 11). Activation of GDH by 2-aminobicyclo-(2,2,1)-heptane-2-carboxylic acid (BCH), a leucine analog, has been extensively used to probe its role in insulin secretion (12, 13). Recent studies suggest that beyond its traditional role, forming α-ketoglutarate, glutamate metabolism may give rise to other metabolites that influence insulin secretion (14, 15).

This study demonstrated that BCH induces biphasic insulin secretion in a manner similar to that of glucose. Subsequently, we examined the metabolic pathways activated downstream of GDH in β-cells. Using a metabolomics approach, we identified a pathway that had not previously been implicated in β-cell stimulus-secretion coupling: the BCH-driven conversion of glutamate into proline. To determine whether proline synthesis plays a role in the biphasic insulin response, we employed a combination of insulin secretion assays, mitochondrial activity measurements, and metabolomics in clonal β-cells or human islets. By exploring this previously unrecognized pathway, our study provides new insights into the metabolic regulation of insulin secretion.

## Results

### Effects of glucose and BCH on insulin secretion

**Figure 1:**
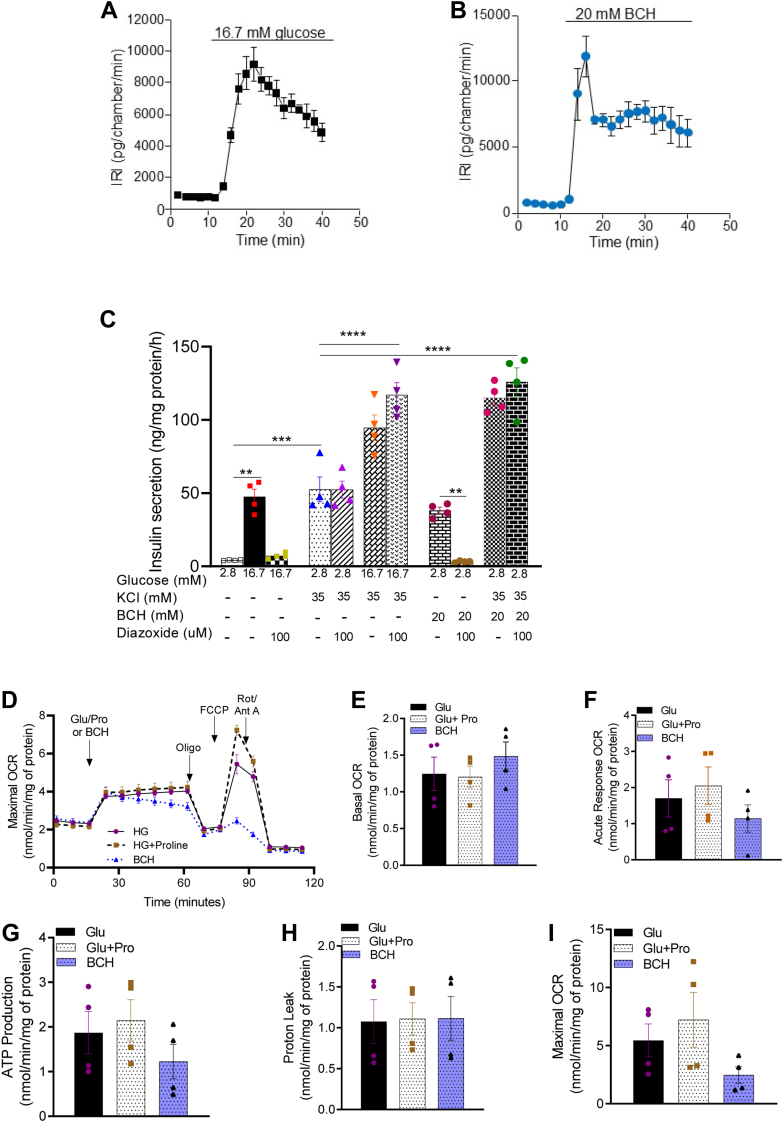
**Effects of glucose and 2-aminobicyclo-(2,2,1)-heptane-2-carboxylic acid (BCH) on insulin secretion and mitochondrial respiration in INS-1 832/13 cells.** Effects of glucose (*A*) and BCH (*B*) on dynamic insulin secretion in INS1-832/13 cells. Immunoreactive insulin (IRI) was measured at 2 min intervals. Effects of diazoxide and KCl on glucose- and BCH-induced insulin secretion (*C*). Effects of glucose, BCH, and proline on mitochondrial oxygen consumption rate (OCR). OCR was measured by Seahorse (XF24) in INS-1 832/13 β-cells (*D*). Mitochondrial activity parameters; basal respiration (*E*), acute response (*F*), ATP production (*G*), proton leak (*H*), and maximal OCR (*I*). Values are mean ± SEM (n = 4, independent experimental replicates). One-way ANOVA followed by Tukey *post hoc* test was used for statistical analysis. ∗∗*p* < 0.01; ∗∗∗*p* < 0.001; ∗∗∗∗*p* < 0.0001.

Both glucose (16.7 mM) and BCH (20 mM) triggered robust, biphasic insulin secretion in INS-1 832/13 cells (Fig. 1). The insulin response to glucose peaked at 12 min, while BCH-induced secretion reached its peak at 8 min, indicating a slightly faster onset for BCH. Following these initial peaks, insulin release gradually stabilized at comparable levels for both stimuli. These findings are consistent with earlier reports demonstrating that BCH activates GDH, which enhances insulin secretion despite not being directly metabolized (16).

To further investigate the potential role of BCH in the metabolic amplification pathway, we performed experiments using diazoxide, a K_ATP_ channel opener that inhibits glucose-induced depolarization, and KCl that directly depolarizes the plasma membrane. INS-1 832/13 cells were treated with 100 μM diazoxide and/or 35 mM KCl for 1 h in the presence of 2.8 and 16.7 mM glucose and BCH, and insulin secretion was measured. As shown in Fig. 1*C*, diazoxide treatment abrogated insulin secretion stimulated by both glucose (16.7 mM) and BCH (20 mM). When KCl was added, a rise in glucose from 2.8 to 16.7 mM enhanced insulin secretion regardless of whether diazoxide was present or not; this is the well-known K_ATP_-independent or amplifying effect of glucose (17). BCH enhanced insulin secretion to the same extent as 16.7 mM glucose when the plasma membrane was depolarized by KCl and the K_ATP_ channel was kept open by diazoxide. This observation is consistent with a role of BCH both in the triggering and amplifying pathway of insulin secretion (7, 17, 18).

### Effects of glucose and BCH on mitochondrial function

Mitochondria are crucial for linking glucose metabolism to insulin secretion in β-cells *via* ATP production. To evaluate the impact of BCH on mitochondrial metabolism, we measured the mitochondrial oxygen consumption rate (OCR) under three conditions: baseline (2.8 mM glucose), 16.7 mM glucose, and 20 mM BCH (Fig. 1*D*). Several parameters, including basal respiration, acute response, ATP-linked respiration, proton leak, and maximal OCR, were assessed to understand the differential effects of glucose and BCH (Fig. 1, *E*–*I*).

Both glucose and BCH significantly increased OCR, indicating enhanced mitochondrial activity. However, BCH’s effect was transient compared to that of glucose, which induced a sustained increase in OCR. This transient response to BCH may be due to rapid substrate depletion, given the role of GDH in converting glutamate into α-ketoglutarate. In contrast, glucose appears to provide a more continuous substrate supply to fuel the TCA cycle and maintain mitochondrial respiration. This observation underscores the importance of substrate availability in sustaining mitochondrial respiration, particularly in β-cells, where nutrient supply directly governs respiration rate (19).

**Figure 2:**
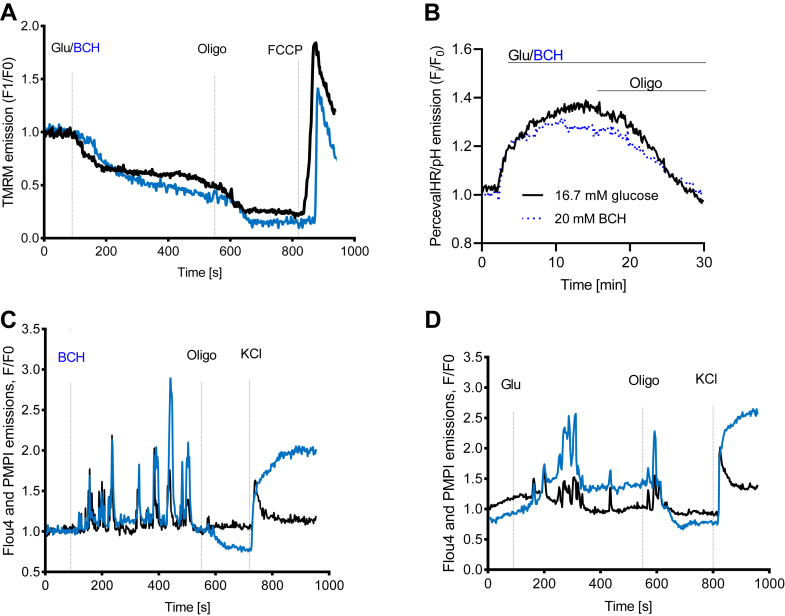
**Effects of glucose and 2-aminobicyclo-(2,2,1)-heptane-2-carboxylic acid (BCH) on mitochondrial membrane potential, ATP/ADP ratio, and ion dynamics in INS-1 832/13 cells.** Effects of glucose (Glu) and BCH on mitochondrial membrane potential (*A*), cytosolic ATP/ADP ratio (*B*), as well as plasma membrane potential (*blue*) and calcium oscillations (*black*) (*C* and *D*) in INS-1 832/13 cells. Values are mean ± SEM (n = 4 independent experimental replicates). FCCP, carbonyl cyanide-p-trifluoromethoxyphenylhydrazone; oligo, oligomycin.

Mitochondrial membrane potential (ΔΨm), a key indicator of mitochondrial activity, reflects electron transport, that is, the proton motive force. We measured ΔΨm with tetramethylrhodamine methyl ester (TMRM) (Fig. 2*A*). ΔΨm was hyperpolarized by both glucose and BCH, which suggests an increased proton motive force contributing to enhanced ATP production. To explore this, we measured the cytosolic ATP/ADP ratio in INS-1 832/13 cells treated with glucose or BCH, using the genetically encoded PercevalHR probe (Fig. 2*B*). Both glucose and BCH raised the cytosolic ATP/ADP ratio in β-cells. Since β-cells primarily generate ATP through oxidative phosphorylation (OXPHOS) (20), the increased ΔΨm and cytosolic ATP/ADP ratio following BCH treatment and GDH activation likely stem from enhanced mitochondrial OXPHOS.

### Effects of glucose and BCH on plasma membrane potential and cytosolic Ca^2+^ levels

In Figure 2, *C* and *D*, a representative trace of INS-1 832/13 cells demonstrates the use of proprietary bis-oxonol membrane potential indicator (PMPI) fluorescence to monitor membrane potential (Vm) changes following the administration of 16.7 mM glucose or 20 mM BCH. Both stimuli induced plasma membrane depolarization, characterized by spikes indicative of Vm bursting. The introduction of 6 μM oligomycin resulted in the cessation of plasma membrane depolarizations, likely due to halted ATP production and the subsequent reactivation of K_ATP_ channel. Plasma membrane depolarization leads to the influx of Ca^2+^ through voltage-gated Ca^2+^ channels, initiating the process of exocytosis. Consequently, we measured cytosolic Ca^2+^ concentrations in response to 16.7 mM glucose or 20 mM BCH in INS-1 832/13 cells. These measurements were performed simultaneously with the PMPI recordings, as depicted in Figure 2, *C* and *D*, and superimposed on the PMPI traces. Clearly, the levels of cytosolic Ca^2+^ were correlated with the increased Vm and the associated membrane potential spikes, irrespective of whether glucose or BCH served as the stimulus.

### Effects of glucose and BCH on cellular metabolites

**Figure 3:**
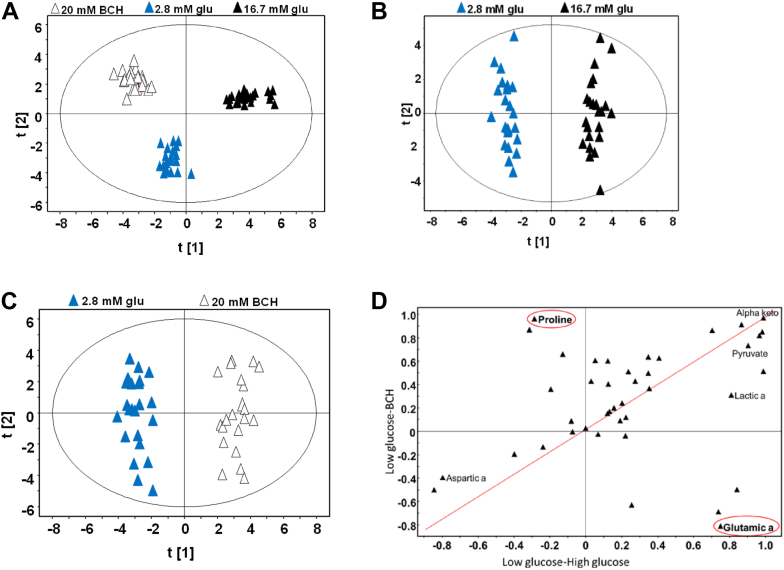
**Orthogonal projections to latent structures–discriminant analysis (OPLS-DA) of metabolic responses to glucose and 2-aminobicyclo-(2,2,1)-heptane-2-carboxylic acid (BCH) in INS-1 832/13 cells.** OPLS-DA based on the metabolite pattern in the three conditions (*A*). Score of the OPLS-DA models describing metabolic alterations between INS-1 832/13 cells exposed to 2.8 and 16.7 mM glucose (Glu; *B*) or 2.8 mM glucose and 20 mM BCH (*C*). Glucose- and BCH-specific metabolite responses in INS-1 832/13 cells (*D*); a two-dimensional correlation loading plot was created from the loading vectors of the OPLS-DA models. Metabolites found on the diagonal, indicated by *red* line, are regulated in a similar fashion in glucose- and BCH-stimulated cells. The opposite diagonal shows an inverse relationship in metabolite responses. n = 3 independent experimental replicates.

Given the similarities in mitochondrial and plasma membrane responses to glucose and BCH, we conducted a metabolomics analysis to uncover potential differences in their metabolic effects. Metabolite profiles were compared at 6 min (triggering phase) and 20 min (amplifying phase) poststimulation with glucose or BCH (Fig. 3).

Initially, we compared the similarities (shared regulation) and differences (unique regulation) in metabolite profiles in cells exposed to 2.8 and 16.7 mM glucose and 20 mM BCH. Using orthogonal projections to latent structures discriminant analysis (OPLS-DA), we achieved classification of samples based on their distinct metabolite patterns under these three conditions (Fig. 3*A*). This classification indicated that glucose and BCH generate distinct metabolic responses despite the similar biphasic pattern of insulin secretion observed under both conditions.

Next, we compared the metabolic effects of stimulation with 16.7 mM glucose and 20 mM BCH against the baseline condition of 2.8 mM glucose using separate OPLS-DA models (Fig. 3, *B* and *C*). Again, the models demonstrated distinct clustering of samples according to the stimulatory conditions, highlighting the capability of OPLS-DA to differentiate between metabolic responses induced by glucose and BCH. Importantly, the degree of metabolic change was similar when comparing the effects of glucose and BCH relative to the basal state, as revealed by the shared and unique structures plot (Fig. 3*D*). This plot illustrates metabolite alterations along two axes, with the x-axis representing changes due to glucose and the y-axis representing changes due to BCH. This layout facilitates the identification of metabolites regulated similarly by both stimuli or uniquely by one condition.

One notable finding was the differential regulation of glutamate, which increased in response to glucose but decreased upon BCH stimulation. In contrast, α-ketoglutarate levels rose similarly under both conditions, suggesting that GDH activation by BCH contributes to α-ketoglutarate production in a manner akin to glucose stimulation. These results imply that, under these experimental conditions, GDH functions anaplerotically, directing carbon flux into the TCA cycle. Furthermore, increased levels of fumarate, malate, pyruvate, and citrate under both glucose and BCH stimulation suggest enhanced TCA cycle activity linked to GDH activation. The reduction in glutamate levels with BCH could be attributed to its consumption *via* other metabolic pathways not fully elucidated in this study.

Additionally, we observed a marked increase in proline levels when cells were stimulated with BCH but not with glucose (Fig. 3), suggesting a potential role for proline in the amplifying pathway. The differential effects on α-ketoglutarate and proline levels prompted further exploration of the potential link between these metabolites. Proline can be synthesized from glutamate *via* proline 5-carboxylate (P5C) synthase, a rate-limiting enzyme that uses NADPH as a cofactor for the reductive synthesis of proline. Conversely, P5C synthase can also operate in reverse, generating nucleotides considered potential coupling factors in insulin secretion (21).

### ALDH18A1 silencing in INS-1 832/13 cells and islets

To manipulate the proline synthesis pathway, we silenced *ALDH18A1*, the gene encoding P5C synthase, achieving a knockdown efficiency of 76 to 80% in INS-1 832/13 cells and 62 to 68% and 56% in rat and human islets at the mRNA levels, respectively (Fig. S1). In addition, protein expression of P5C synthase was reduced by 95% following *ALDH18A1* knockdown (Figs. S1, S2). To evaluate the transfection efficiency, we cotransfected *ALDH18A1* siRNA with BLOCK-iT Alexa Fluor Red Fluorescent Control and assessed the percentage of transfected cells using flow cytometry. This analysis revealed a high transfection efficiency, with nearly 90% of the cells transfected (Fig. S3). With these models in place, we then investigated the functional impact of *ALDH18A1* knockdown on insulin secretion in INS-1832/13 cells and rat/human islets exposed to glucose or BCH.

### Effects of ALDH18A1 silencing on insulin secretion

**Figure 4:**
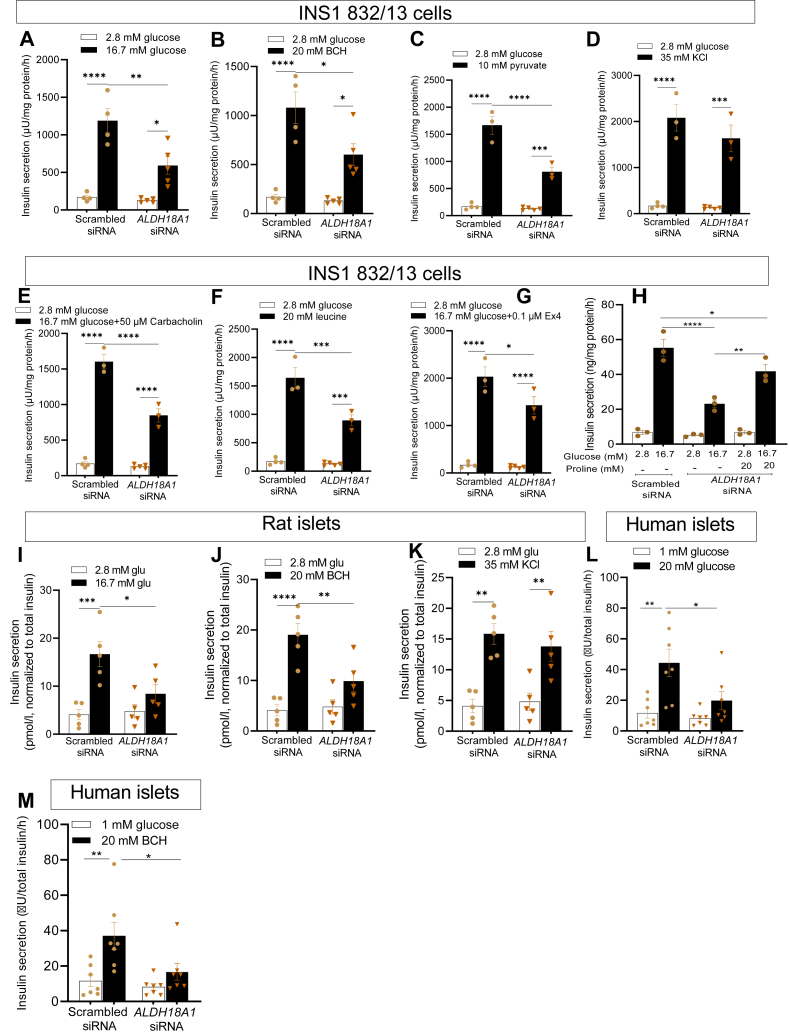
**Impact of ALDH18A1 knockdown on stimulated insulin secretion in INS-1 832/13 cells, rat islets, and human islets.** Effect of *ALDH18A1* knockdown on glucose- (Glu; *A*), 2-aminobicyclo-(2,2,1)-heptane-2-carboxylic acid (BCH)- (*B*), pyruvate- (*C*), KCl- (*D*), carbachol- (*E*), leucine- (*F*), and Exendin-4- (*G*) stimulated insulin secretion in INS1 832/13 cells. Effects of proline on insulin secretion in *ALDH18A1* knockdown INS1 832/13 cells (*H*). Effect of *ALDH18A1* knockdown on glucose- (*I*), BCH- (*J*), and KCl- (*K*) stimulated insulin secretion in rat islets as well as glucose- (*L*) and BCH- (*M*) stimulated insulin secretion in *ALDH18A1*-deficient human islets. Values are mean ± SEM (n = 4–7 independent experimental replicates). One-way ANOVA followed by Tukey *post hoc* test was used for statistical analysis. ∗*p* < 0.05; ∗∗*p* < 0.01; ∗∗∗*p* < 0.001; ∗∗∗∗*p* < 0.0001.

Silencing *ALDH18A1*, which reduces P5C synthase expression, impaired insulin secretion stimulated by glucose, BCH, pyruvate, carbachol, leucine, and exendin-4 but had no effect on KCl-stimulated insulin secretion in INS-1 832/13 cells (Fig. 4, *A*–*G*). To confirm the role of proline depletion for the abrogation of insulin secretion, proline (20 mM) was added along with 16.7 mM glucose to *ALDH18A1* knockdown cells for 1 h (Fig. 4*H*). Indeed, this treatment restored insulin secretion in *ALDH18A1* knockdown cells, indicative of a functional rescue, confirming that the reduction in insulin secretion upon *ALDH18A1* knockdown is directly linked to a deficit in proline metabolism. Furthermore, the rescue of insulin secretion by exogenous proline underscores the critical role of proline in supporting mitochondrial activity and β-cell stimulus-secretion coupling. Similarly, in both rat (Fig. 4, *I*–*K*) and human (Fig. 4, *L* and *M*) islets, *ALDH18A1* knockdown led to reduced insulin secretion in response to glucose or BCH stimulation, while KCl-induced secretion remained unaffected. These findings indicate that P5C synthase is a crucial component of the metabolic pathways driving stimulus-secretion coupling in β-cells.

### Effects of ALDH18A1 silencing on mitochondrial function

**Figure 5:**
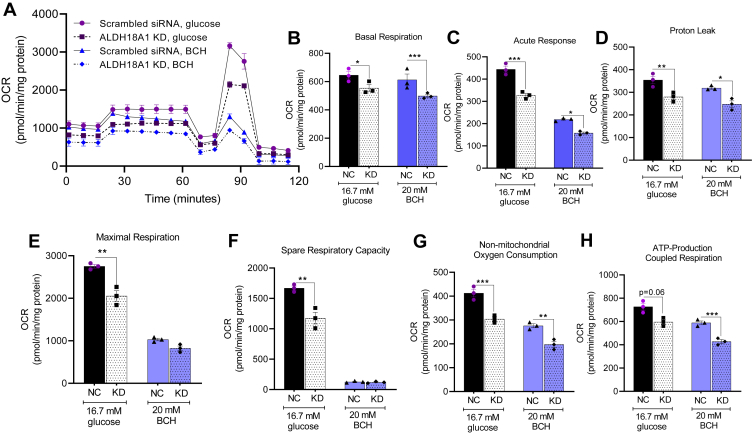
**Effects of glucose and 2-aminobicyclo-(2,2,1)-heptane-2-carboxylic acid on mitochondrial oxygen consumption rate in *ALDH18A1*-silenced (knock down; KD) INS-1 832/13 cells.** OCR was measured by Seahorse XF24 in *ALDH18A1* KD INS-1 832/13 cells (*A*). Mitochondrial activity parameters; basal respiration (*B*), acute response (*C*), proton leak (*D*), maximal OCR (*E*), spare respiratory capacity (*F*), non-mitochondrial OCR (*G*)*,* and ATP production (*H*). Values are mean ± SEM (n = 3 independent experimental replicates). Unpaired Student's *t* test was used for statistical analysis. ∗*p* < 0.05; ∗∗*p* < 0.01; ∗∗∗*p* < 0.001.

P5C synthase plays a role in mitochondrial function and organization of respiratory complexes. Given that BCH enhances OXPHOS and stimulates proline production in clonal β-cells, we hypothesized that the increased OXPHOS following BCH treatment is linked to elevated proline levels. We measured mitochondrial OCR in response to 20 mM proline (Fig. 1), as well as in *ALDH18A1*-silenced INS-1 832/13 cells treated with 16.7 mM glucose or 20 mM BCH (Fig. 5). Adding proline potentiated mitochondrial OCR in response to glucose, indicating a positive effect on mitochondrial respiration (Fig. 1). In contrast, silencing *ALDH18A1* significantly reduced mitochondrial OCR upon stimulation with glucose or BCH (Fig. 5), suggesting that the reduction in OCR is due to decreased proline formation.

**Figure 6:**
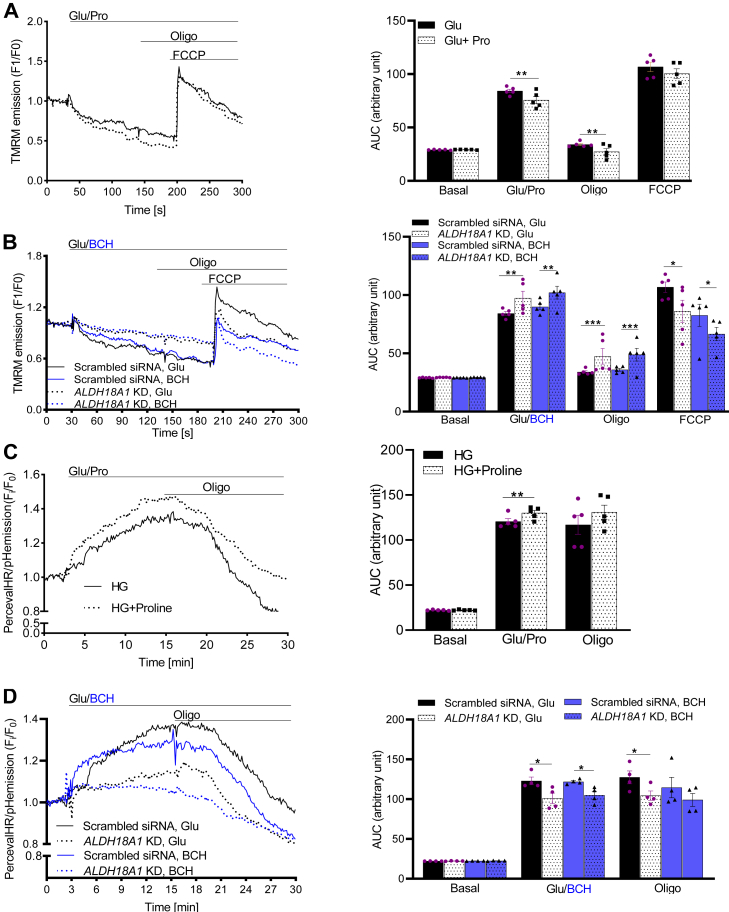
**Effects of proline and *ALDH18A1*-silencing (knockdown; KD) on mitochondrial inner membrane potential and cytosolic ATP/ADP ratio in INS-1 832/13 cells.** Effect of proline (*A*) and *ALDH18A1* KD (*B*) on mitochondrial membrane potential in INS-1 832/13 cells. Effect of proline (*C*) and *ALDH18A1* KD (*D*) on cytosolic ATP/ADP ratio in INS-1 832/13 cells. Areas under the curves are shown in the *left* columns. Values are mean ± SEM (n = 5 independent experimental replicates). Unpaired Student's *t* test was used for statistical analysis. ∗*p* < 0.05; ∗∗*p* < 0.01; ∗∗∗*p* < 0.001. Glu, glucose.

We then examined mitochondrial membrane potential under the same condition. Proline enhanced glucose-induced hyperpolarization of Δψm in control cells (Fig. 6*A*), but *ALDH18A1* silencing reduced this hyperpolarization when the clonal β-cells were stimulated by glucose or BCH (Fig. 6*B*).

To further investigate the impact on ATP production, we measured the cytosolic ATP/ADP ratio. Proline significantly increased this ratio in control cells (Fig. 6*C*), whereas *ALDH18A1* silencing diminished the glucose- or BCH-induced elevation in ATP/ADP ratio (Fig. 6*D*). These findings indicate that BCH-induced increases in ATP/ADP ratios are partially due to proline formation.

### Effects of ALDH18A1 silencing on plasma membrane potential and cytosolic Ca^2+^ levels

Since BCH treatment led to increased proline production, plasma membrane depolarization, and elevated intracellular Ca^2+^ levels, we explored whether these effects were directly linked to proline. We measured Vm and cytosolic Ca^2+^ levels in response to 20 mM proline in control and *ALDH18A1*-silenced cells treated with 16.7 mM glucose or 20 mM BCH.

**Figure 7:**
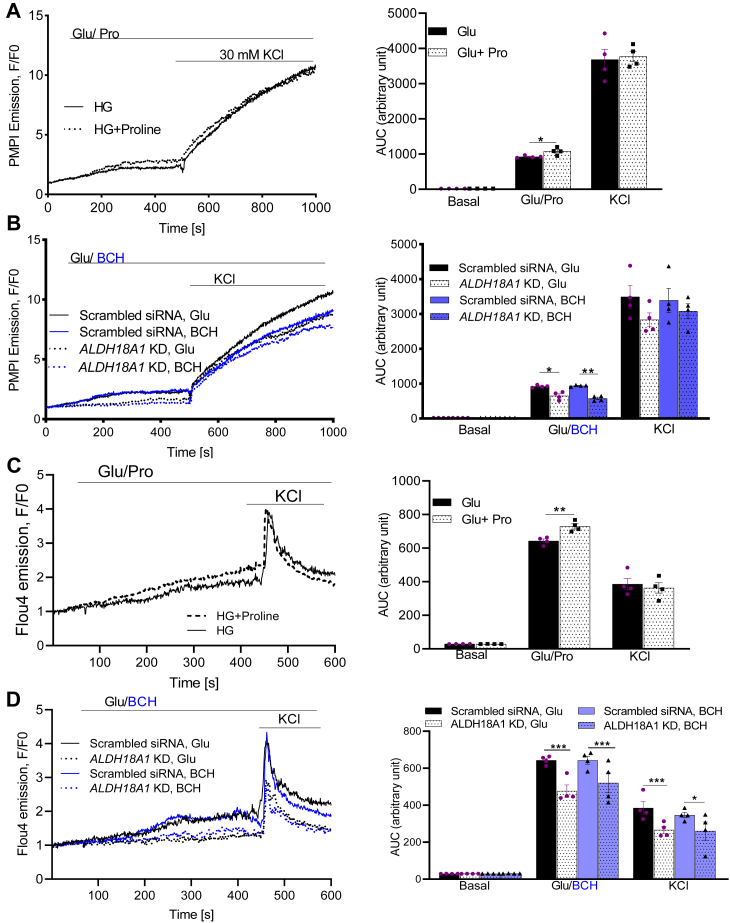
**Effects of proline and *ALDH18A1*-silencing (knock down; KD) on plasma membrane potential and cytosolic Ca^2+^ levels in INS-1 832/13 cells.** Effect of proline (*A*) and *ALDH18A1* KD (*B*) on plasma membrane potential in INS-1 832/13 cells. Effect of proline (*C*) and *ALDH18A1* KD (*D*) on cytosolic Ca^2+^ levels in INS-1 832/13 cells. Areas under the curves are shown in the *left* columns. Values are mean ± SEM (n = 4 independent experimental replicates). Unpaired Student's *t* test was used for statistical analysis. ∗*p* < 0.05; ∗∗*p* < 0.01; ∗∗∗*p* < 0.001. Glu, glucose.

Proline enhanced the glucose-induced elevation in Vm (Fig. 7*A*) and cytosolic Ca^2+^ levels (Fig. 7*C*) in control cells. However, silencing of *ALDH18A1* diminished the increase in Vm (Fig. 7*B*) and cytosolic Ca^2+^ levels (Fig. 7*D*) in response to glucose or BCH. The depolarizing effect of proline, which primarily is cotransported with Na^+^, resulted in increased plasma membrane depolarization and influx of Ca^2+^ (22).

### Metabolomics analysis of proline effects in INS-1 832/13 cells

**Figure 8:**
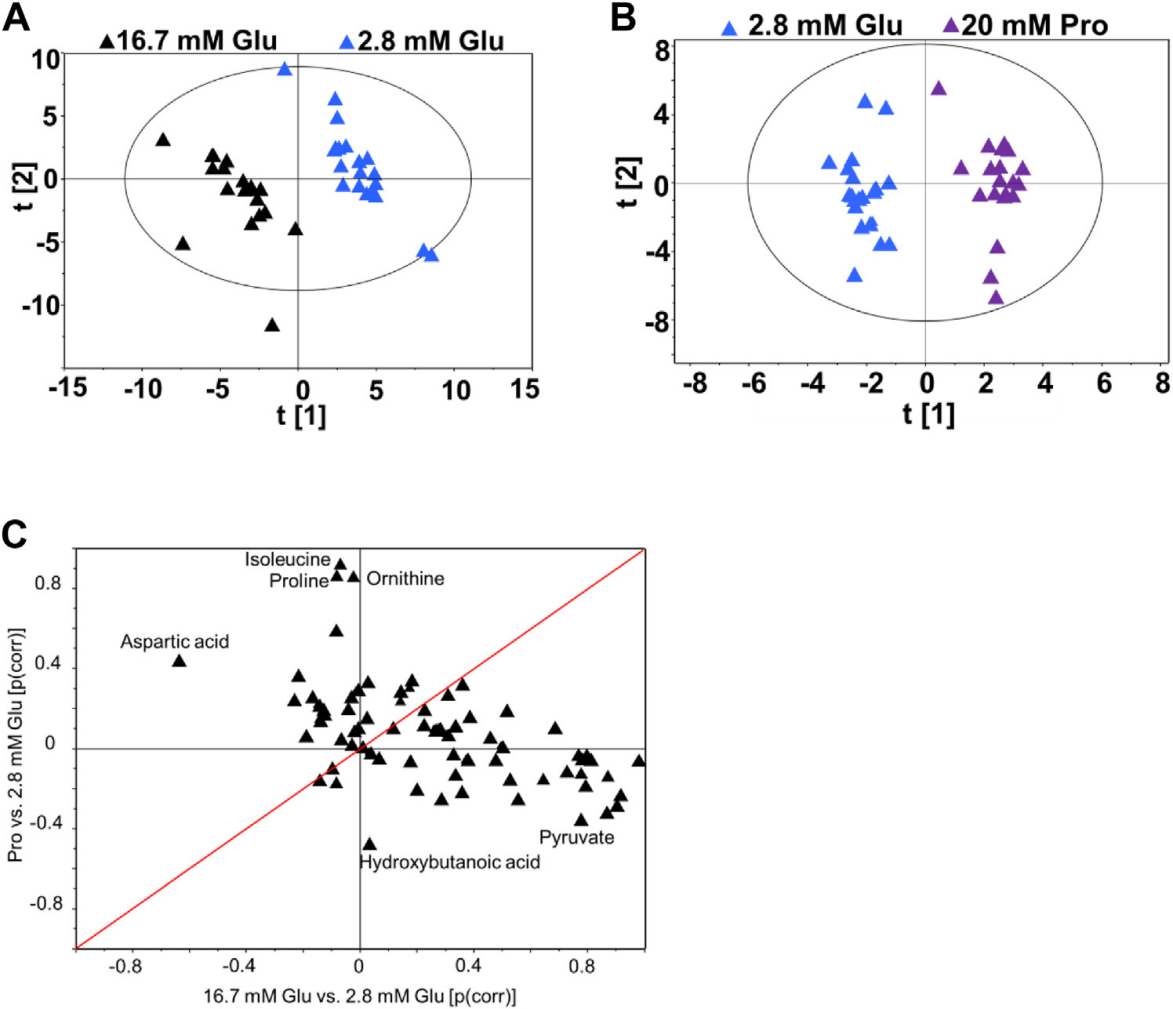
**Multivariate analysis of metabolic responses to glucose and proline in INS-1 832/13 cells***. A*, principal component analysis (PCA) plot of 2.8- and 16.7-mM glucose-exposed INS-1 832/13 cells. *B*, orthogonal projections to latent structures–discriminant analysis (OPLS-DA) plot of cells treated with and without proline. *C*, shared and unique structures (SUS) plot of the correlation of proline (with and without) and glucose levels (2.8 and 16.7 mM) in cells. n = 3 independent experimental replicates.

Metabolomics analysis revealed significant differences in the cellular metabolome under varying glucose and proline conditions. Principal component analysis demonstrated distinct clustering of cells exposed to 2.8 and 16.7 mM glucose (Fig. 8*A*). OPLS-DA further highlighted differences in the metabolome between cells treated with and without proline (Fig. 8*B*).

To identify unique and shared metabolic changes, we constructed separate OPLS-DA models for proline and glucose-stimulated cells, using 2.8 mM glucose as a common control. The combined shared and unique structures plot (Fig. 8*C*) revealed that proline treatment significantly altered levels of isoleucine, ornithine, aspartic acid, and nicotinamide. Conversely, hydroxybutanoic acid and pyruvate were notably altered in response to glucose levels.

## Discussion

In this study, we explored metabolic pathways involved in β-cell stimulus-secretion coupling by comparing the effects of glucose and BCH, a leucine analog and GDH activator, on β-cell and islet function. Our findings revealed a novel role of proline metabolism in the pancreatic β-cell response to BCH, which may contribute to the metabolic regulation of insulin secretion.

Our data showed that both glucose and BCH induced biphasic insulin secretion in INS-1 832/13 cells. This was associated with increased mitochondrial activity, ATP/ADP ratios, plasma membrane depolarization, and cytosolic Ca^2+^ influx. The observations align with earlier reports on the role of GDH activation in insulin secretion *via* enhanced ATP production and subsequent Ca^2+^ influx (16, 23, 24, 25). Interestingly, while glucose and BCH shared similar effects on insulin secretion and mitochondrial function, only stimulation by BCH led to a significant elevation in proline levels. This indicates a stimulus-specific activation of proline metabolism, which, to our knowledge, has not been previously associated with β-cell function and control of insulin secretion.

Previous studies have highlighted the importance of amino acid metabolism in β-cells (26, 27), yet the role of proline remained unexplored. Our data show that BCH-stimulated proline synthesis occurs through ALDH18A1, the enzyme responsible for converting glutamate into proline *via* P5C. Proline contributed to mitochondrial activity by replenishing TCA cycle intermediates through its metabolism into glutamate and subsequently α-ketoglutarate. This process will ensure a steady supply of TCA intermediates, which support continuous mitochondrial ATP production essential for β-cell energy demands during insulin secretion (28). Moreover, proline metabolism is closely linked to maintaining cellular redox balance. By cycling between its oxidized and reduced forms, proline serves as a redox buffer, facilitating electron transfer and stabilizing mitochondrial function under metabolic stress (29). This dual role of proline in fueling the TCA cycle and supporting redox homeostasis underscores its importance in linking nutrient metabolism to enhanced β-cell stimulus-secretion coupling.

Silencing *ALDH18A1* significantly abrogated insulin secretion and mitochondrial function in response to both glucose and BCH. These effects were associated with reduced ATP/ADP ratios and lower cytosolic Ca^2+^ levels, suggesting a role for proline in maintaining the energy status and hence stimulus-secretion coupling in β-cells. The involvement of proline in BCH-induced insulin secretion highlights the ability of β-cells to utilize alternate metabolic pathways beyond the canonical glycolytic and TCA cycle routes. The observed increase in proline levels following BCH treatment suggests that proline metabolism may complement glucose metabolism, enabling β-cells to respond to specific metabolic cues, such as GDH activation. This perspective aligns with the emerging understanding that β-cell metabolism integrates pathways of glucose, lipids, and amino acids, offering potential insights for diabetes therapies targeting β-cell function (27).

A key strength of this study is the integration of metabolic, mitochondrial, and functional assays to investigate the role of proline in β-cell stimulus-secretion coupling. By employing INS-1 832/13 cells, rat islets, and human islets, we confirmed the relevance of our findings across multiple models. However, there are limitations to acknowledge. First, while INS-1 832/13 cells are widely used as a model for β-cell function, their metabolic profile does not entirely reflect that of primary human β-cells, including lower glucose sensitivity, altered metabolic coupling, and metabolism serving active cellular replication. These differences can result in attenuated calcium fluxes in response to glucose and BCH stimulation when compared to native mouse or human β-cells. To ensure translational relevance, future studies should aim to replicate these findings using a more diverse range of human islets from different donors. Second, although we measured steady-state metabolite levels, this approach does not capture dynamic metabolic fluxes, limiting our understanding of the temporal changes in metabolic pathways. Future research incorporating fluxomics and isotopic tracing techniques could enable more precise mapping of these pathways, particularly the contributions of proline and related metabolites. Third, while BCH is an established tool in islet research, it is not entirely specific. As a leucine analog, BCH can interact with multiple metabolic pathways, potentially affecting not only insulin secretion but also broader aspects of amino acid metabolism. Its ability to influence enzymes involved in branched-chain amino acid transamination and catabolism may lead to off-target effects, complicating the interpretation of results. Therefore, while BCH remains valuable for studying insulin secretion, its lack of complete specificity should be considered when drawing conclusions from studies utilizing this compound. Finally, our focus was on *ALDH18A1*-mediated proline synthesis; other enzymes and pathways within the proline and glutamate network may also be involved in β-cell function. Therefore, further genetic and pharmacological studies are warranted to elucidate the broader implications of these pathways in β-cell metabolism and insulin secretion.

## Conclusion

Our study uncovers a potential role for the conversion of glutamate to proline in regulating insulin secretion in pancreatic β-cells, particularly in the context of GDH activation by BCH. This pathway appears to contribute to mitochondrial function, calcium homeostasis, and energy production, which are important for insulin exocytosis. While these findings enhance our understanding of β-cell metabolism, further studies are needed to fully elucidate the clinical implications. Nonetheless, targeting proline metabolism might offer a complementary approach for enhancing β-cell function in diabetes, alongside established glucose-focused therapies.

## Experimental procedures

### Cell culture and rat/human pancreatic islets

#### INS-1 832/13 cells

Clonal INS-1 832/13 cells were cultured in RPMI-1640 medium supplemented with 11.1 mM glucose, 10% fetal bovine serum (FBS), 10 mM Hepes, 2 mM glutamine, 1 mM sodium pyruvate, and 50 μM β-mercaptoethanol. The cells were maintained at 37 °C in a 5% CO_2_ incubator.

#### Rat islets

Rat pancreatic islets were isolated using a collagenase digestion method as described previously (30). Postisolation, islets were cultured in RPMI 1640 with GlutaMax (Gibco-BRL, DK), supplemented with 10% FBS. The animal procedures were approved by the local animal ethics committee in Malmö/Lund, Sweden (2931/20).

#### Human islets

Human pancreatic islets were procured from the Nordic Network for Clinical Islet Transplantation at Uppsala University, Sweden, *via* the Human Tissue Laboratory at Lund University Diabetes Centre. The islets were cultured in RPMI 1640 with GlutaMax (Gibco-BRL, DK), supplemented with 10% FBS, 100 U/ml penicillin, and 100 μg/ml streptomycin. Clinical characteristics of the islet donors are detailed in Table S1. All procedures involving human islets were approved by the ethical committees in Uppsala and Malmö/Lund, Sweden (2019-00357) and abide by the Declaration of Helsinki principles.

### ALDH18A1 knockdown in INS-1 832/13 cells and rat/human islets

#### INS-1 832/13 cells

To silence *ALDH18A1* expression, INS-1 832/13 cells were seeded at a density of 2 × 10^∧^5 cells/cm^2^ in 24-well plates and cultured overnight. *ALDH18A1* expression was silenced by transfecting cells with 50 nM siRNAs specific to rat *ALDH18A1* (Thermo Fisher Scientific, Cat. 4390771) or a scrambled siRNA control using Lipofectamine RNAiMAX Reagent (Thermo Fisher Scientific). Cells were transfected for 72 h prior to experimentation.

#### Rat and human islets

Rat or human islets (200–300 islets per condition) were transfected with 50 nM *ALDH18A1* siRNAs (rat: Thermo Fisher Scientific; human: Thermo Fisher Scientific; Cat. 4427038 (s11633)) using Lipofectamine RNAiMAX. After 24 h, islets were retransfected for an additional 24 h, and functional assays were performed 72 h post-transfection.

### Evaluating of the transfection efficiency using BLOCK-iT Alexa Fluor Red Fluorescent Control

INS-1 832/13 cells were seeded at a density of 2 × 10^∧^5 cells/cm^2^ in 24-well plates and cultured overnight. Transfection was performed using *ALDH18A1* siRNA (50 nM) in combination with BLOCK-iT Alexa Fluor Red Fluorescent Control (50 nM; Thermo Fisher Scientific, Cat. #14750100) and Lipofectamine RNAiMAX Reagent. The transfection efficiency, defined as the percentage of transfected cells, was assessed 24 h post-transfection *via* flow cytometry.

### RNA isolation and quantitative real-time PCR

Total mRNA was isolated with an RNA purification kit (Qiagen) and then reverse transcribed into cDNA using the RevertAid First-Strand cDNA Synthesis Kit (Thermo Fisher Scientific), following the manufacturer’s protocols. Quantitative Real-Time PCR was conducted with TaqMan gene expression assays for *ALDH18A1* (Thermo Fisher Scientific, # Rn01411415_m1 (for rat) and # Hs00913259_m1 (for human)) and the housekeeping gene HPRT1 (Thermo Fisher Scientific, CAT# 4331182). Gene expression levels were calculated using the comparative Ct method, where target expression is reported as 2-ΔΔCt, with HPRT1 serving as the reference gene.

### Western blotting

Seventy-two hours post-transfection, INS-1 832/13 cells were washed with PBS and lysed using RIPA buffer supplemented with a protease inhibitor cocktail for 20 min on ice. The lysates were centrifuged at 14,000*g* for 15 min, and the protein concentration was quantified using a bicinchoninic acid (BCA) assay kit (Thermo Fisher Scientific). Equal amounts of protein (30 μg) were separated *via* SDS-PAGE using Mini-PROTEAN TGX Stain-Free Protein Gels (Cat. #4568124, BioRad) and transferred to polyvinylidene difluoride membranes (Cat. #1704156, BioRad). The membranes were blocked with a 5% (wt/vol) dry milk solution for 1 h and then incubated overnight at 4 °C with primary antibodies against P5C synthase (Proteintech, Cat. #17719-1-AP) or β-actin (Cell Signaling, Cat. #3700). Secondary antibodies used were anti-rabbit IgG, HRP-linked (Cat. #7074, Cell Signaling) and anti-mouse IgG, HRP-linked (Cat. #7076P2, Cell Signaling). Washing steps were performed with TBS containing 0.1% (vol/vol) Tween-20. To visualize the blots, Clarity Western ECL Substrate (Cat. #1705060, BioRad) was applied to the membranes, and images were captured using a charge-coupled device camera and analyzed with Image Lab software (BioRad). Quantification of protein bands was performed using ImageJ software, with background subtraction and normalization where appropriate.

### Insulin secretion assays

#### Static incubation

Seventy-two hours post-transfection, INS-1 832/13 cells were pre-incubated in secretion assay buffer (114 mM NaCl, 1.2 mM KH_2_PO_4_, 4.7 mM KCl, 1.16 mM MgSO_4_, 2.5 mM CaCl_2_, 100 mM Hepes, 25 mM NaHCO_3_, 0.2% BSA, pH 7.4) with 2.8 mM glucose for 2 h at 37 °C. Cells were then stimulated with 16.7 mM glucose, 20 mM BCH, or 35 mM KCl for 1 h. Insulin was measured in the supernatant and lysed cells using the Rat Insulin ELISA kit (Mercodia), and values were normalized to total protein content (BCA Protein Assay Kit, Thermo Fisher Scientific).

#### Rat/human islets

Four replicates of eight islets per condition were pre-incubated in secretion assay buffer (with 2.8 mM glucose for 2 h for rat islets and 1 mM glucose for 1 h for human islets). The islets were then stimulated with 16.7 or 20 mM glucose (for rat or human islets, respectively), 20 mM BCH, or 35 mM KCl for 1 h. Insulin secretion was measured in the supernatant and total insulin content in lysed islets, with results normalized to total insulin content and protein content, respectively.

#### Dynamic perfusion

Approximately 3 × 10^6^ INS-1 cells were placed in each of the four chambers perfused at 1 ml/min. The cells were perfused with buffer containing 2.8 mM glucose for 10 min, followed by stimulation with 16.7 mM glucose or 20 mM BCH. Samples were collected at 2-min intervals, and insulin levels in the supernatant were measured and normalized to total protein content (31, 32).

### Plasma and mitochondrial membrane potentials

#### Plasma membrane potential (Vm)

A vial from the FLIPR Membrane Potential Assay Kit, component A (Molecular Devices; CAT# R-8042), containing a Vm indicator (PMPI), was reconstituted in 10 ml of distilled water, aliquoted into 1 ml portions, and stored frozen. Cells were seeded (7 × 10^∧^4 cells/cm^2^) on poly-D-lysine–coated 8-well chambered cover glasses and pre-incubated in imaging buffer (135 mM of NaCl, 3.6 mM of KCl, 1.5 mM of CaCl_2_, 0.5 mM of MgSO_4_, 0.5 mM of Na_2_HPO_4_, 10 mM of Hepes, and 5 mM of NaHCO_3_ at a pH of 7.4) with 2.8 mM glucose for 2 h. Just before imaging, cells were loaded with PMPI at 2 μl per 400 μl buffer for 10 min. After capturing baseline PMPI fluorescence in 2.8 mM glucose, fluorescence was recorded with excitation at 543 nm and emission at 565 to 615 nm in response to high glucose (16.7 mM), BCH (20 mM), and KCl (30 mM).

#### Mitochondrial membrane potential (Δψm)

INS-1 cells were placed (7 × 10^∧^4 cells/cm^2^) in poly-D-lysine–coated 8-well chambered cover glasses and pre-incubated for 2 h in imaging buffer with 2.8 mM glucose, 100 nM TMRM (Invitrogen), and 2.5 mM cyclosporin A. Following incubation, the cells were rinsed and then examined by live-cell confocal microscopy in quench mode, where ΔΨm decreases after an initial transient increase in whole-cell fluorescence. Baseline TMRM fluorescence was recorded at 2.8 mM glucose, and additional fluorescence emissions were recorded in response to glucose (16.7 mM), BCH (20 mM), oligomycin (6 μM), and FCCP (4 μM).

### Measurement of cytosolic Ca^2+^ levels

INS-1 cells placed in poly-D-lysine–coated 8-well chambered cover glasses were pre-incubated in imaging buffer with 2.8 mM glucose for 2 h at 37 °C. After 1.5 h of incubation, the cells were loaded with 2.5 μM Fluo-4 AM, along with 0.5 mM and 16 μM BSA, and incubated for an additional 30 min. After incubation, 2 μl of PMPI were added, and cells were imaged. Fluo-4 AM excitation was set at 488 nm, and emission was recorded at 505 nm using a Zeiss LSM510 inverted confocal fluorescence microscope. PMPI was excited at 543 nm and emission detected at >560 nm (33). Baseline Fluo-4 AM and/or PMPI fluorescence was captured at 2.8 mM glucose, followed by fluorescence measurements in response to glucose (16.7 mM), BCH (20 mM), oligomycin (6 μM), and KCl (30 mM).

### Measurement of mitochondrial OCR

Mitochondrial OCR was assessed using a Seahorse Extracellular Flux Analyzer XF24 (Seahorse Bioscience), following the manufacturer’s protocols. Following a 2-h starvation period in 2.8 mM glucose, OCR was measured every 3 min for 90 min in INS-1 832/13 cells under basal conditions and following treatment with glucose (2.8 and 16.7 mM), BCH (20 mM), oligomycin (4 μM), FCCP (4 μM), and rotenone/antimycin A (1 μM). Data were analyzed using Seahorse Wave software (Agilent).

### Measurement of cytosolic ATP/ADP ratio

The cytosolic ATP/ADP ratio was measured using the genetically encoded biosensor Perceval HR and pHRed. Cells seeded onto poly-D-lysine–coated 8-well chambered cover glasses were cotransfected with Perceval HR plasmid and *ALDH18A1* siRNA. After a 1-h starvation in imaging buffer containing 2.8 mM glucose, pHrodo Red AM (1:1000 dilution) was added for 1 h. Perceval HR fluorescence was excited at 490 nm and emission recorded at 535 nm. pHrodo Red AM fluorescence was excited at 560 nm and emission recorded at 585 nm on a Zeiss LSM510 confocal microscope.

### Metabolomics analysis

Metabolomics analysis was performed as previously described (34, 35). Cells were incubated as for insulin secretion assays, then washed with ice-cold PBS and quenched with 400 μl methanol at −80 °C. After scraping and sonication, metabolites were extracted with methanol/water (80/20, v/v) containing stable isotope-labeled internal standards. The extract was dried and derivatized with methoxyamine hydrochloride and MSTFA + 1% trimethylsilyl chloride. Samples were analyzed by gas chromatography/mass spectrometry using an Agilent 7890A GC coupled to an Agilent 5975C VL MSD. Data were processed using MS-DIAL version 4.7 for peak extraction, alignment, deconvolution, and annotation. Metabolite identification was achieved using the Fiehn BinBase DB.

### Statistical analysis

Metabolite peak areas were analyzed using SIMCA 17.0 (Umetrics AB), mean-centered, UV-scaled, and subjected to principal component analysis and OPLS-DA. Data are presented as mean ± SD or mean ± SEM. Normal distribution of data was verified using the Shapiro–Wilk test. Statistical significance was determined using Student’s *t* test or one-way ANOVA followed by Tukey’s *post hoc* test for multiple comparisons.

## Data availability

The data supporting this study are available upon request from the corresponding author.

## Supporting information

This article contains supporting information.

## Conflicts of interest

The authors declare that they have no conflicts of interests with the contents of this article.
