# Supplementary material for: Conversion of glutamate into proline by the leucine analog BCH enhances biphasic insulin secretion in pancreatic β-cells
Source: J Biol Chem. 2025 Mar 26;301(5):108449. doi: 10.1016/j.jbc.2025.108449 (PMC12059337; doi:10.1016/j.jbc.2025.108449)
Supplement: Supporting information [file mmc1.docx]

**Conversion of glutamate into proline by the leucine analog BCH enhances biphasic insulin secretion in pancreatic β-cells**

Sevda Gheibi ^1, *^, Luis Rodrigo Cataldo ^1,2^, Hamidreza Ardalani ^3,4^, Lisa Nocquet ^1^, Peter Spégel ^3^, Susanne G. Straub ^5^, Geoffrey W.G Sharp ^5^, Malin Fex ^1^, Hindrik Mulder ^1^

1. *Unit of Molecular Metabolism, Lund University Diabetes Centre, SE-20213 Malmö, Sweden.*
2. *Novo Nordisk Foundation Center for Basic Metabolic Research, Faculty of Health and Medical Sciences, University of Copenhagen, Copenhagen 2200, Denmark*
3. *Department of Chemistry, Centre for Analysis and Synthesis, Lund University, Lund, Sweden.*
4. *Pharmacokinetics, Dynamics, and Metabolism, Pfizer R&D, Pfizer Inc., Bothell, Washington, USA*
5. *Department of Molecular Medicine, Cornell University, Ithaca, NY 14853, USA*

Running title: BCH boosts glutamate-to-proline conversion, enhancing insulin secretion

* Communicating author:

Sevda Gheibi

Unit of Molecular Metabolism,

Lund University, CRC, 91:11:018

Jan Waldenströmsg 35,

SE-202 13 Malmö, Sweden

Phone: +46 40 391022

Mobile: +46 76 632 27 97

ORCID: 0000-0001-9687-8659

[Sevda.gheibi@med.lu.se](mailto:Sevda.gheibi@med.lu.se)

Table S1. Characteristics of pancreatic islet donors used for assessing insulin secretion

| **Islet preparation** | **1** | **2** | **3** | **4** | **5** | **6** | **7** |
| --- | --- | --- | --- | --- | --- | --- | --- |
| Unique identifier | HTL378 | HTL379 | HTL377 | HTL376 | HTL375 | HTL374 | HTL373 |
| Donor age (years) | 40 | 53 | 54 | 62 | 65 | 44 | 48 |
| Donor sex (M/F) | F | M | M | F | F | M | M |
| Donor BMI (kg/m^2^) | 30.2 | 32.1 | 23.8 | 25.5 | 23.8 | 35.6 | 26.9 |
| Donor HbA_1c_ | 5.4 | 6.1 | 5.7 | 6 | 6.5 | 5.5 | 6.5 |
| Source of islets | Nordic Network for Clinical Islet Transplantation | Nordic Network for Clinical Islet Transplantation | Nordic Network for Clinical Islet Transplantation | Nordic Network for Clinical Islet Transplantation | Nordic Network for Clinical Islet Transplantation | Nordic Network for Clinical Islet Transplantation | Nordic Network for Clinical Islet Transplantation |
| Islet isolation centre | Islet isolation facility in Uppsala | Islet isolation facility in Uppsala | Islet isolation facility in Uppsala | Islet isolation facility in Uppsala | Islet isolation facility in Uppsala | Islet isolation facility in Uppsala | Islet isolation facility in Uppsala |
| Donor history of diabetes? | No | No | No | No | No | No | No |


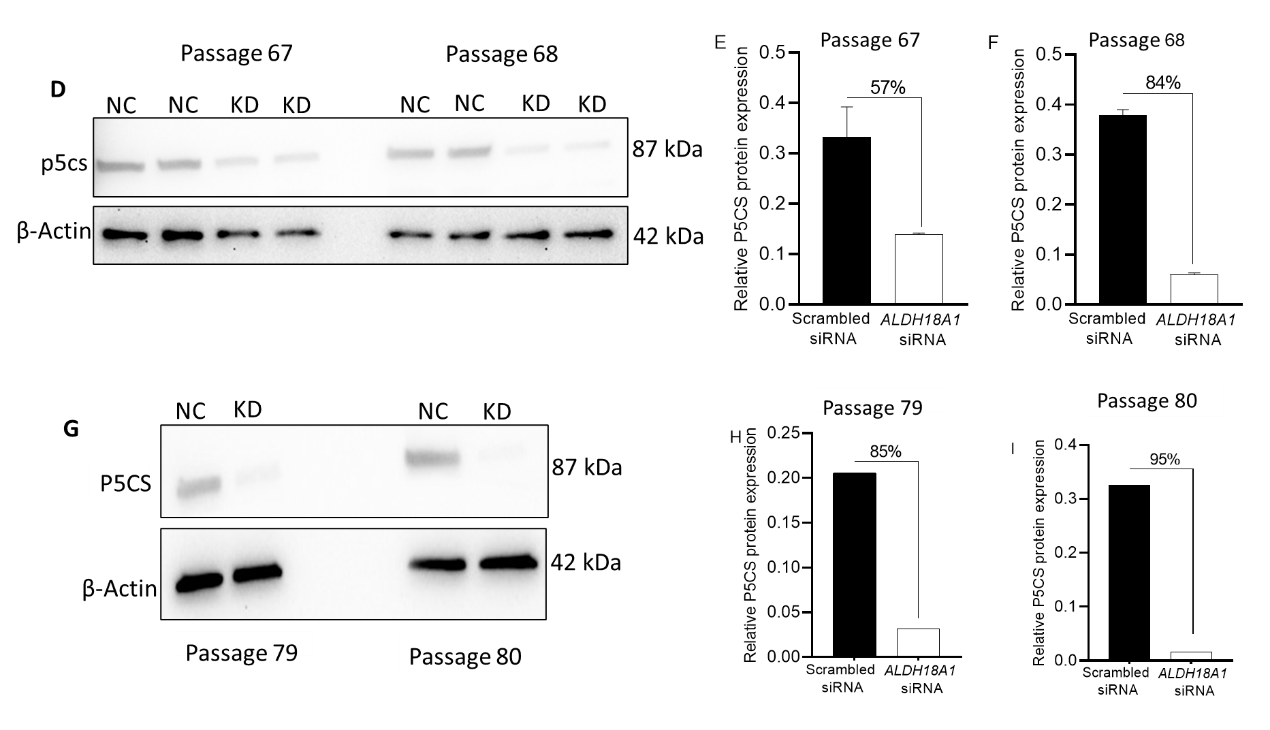


**Supplementary Fig. 1.** mRNA expression of *ALDH18A1* following *ALDH18A1* knockdown in INS-1 832/13 cells (A), rat pancreatic islets (B), and human pancreatic islets (C). Protein expression of P5CS after *ALDH18A1* knockdown in INS-1 832/13 cells at four different passages is shown (D and G), along with their relative expression quantification (E-F and H-I). Protein band quantification was performed using ImageJ software, with background subtraction and normalization where applicable. Data are presented as percentages (n=4-6 independent experimental replicates).


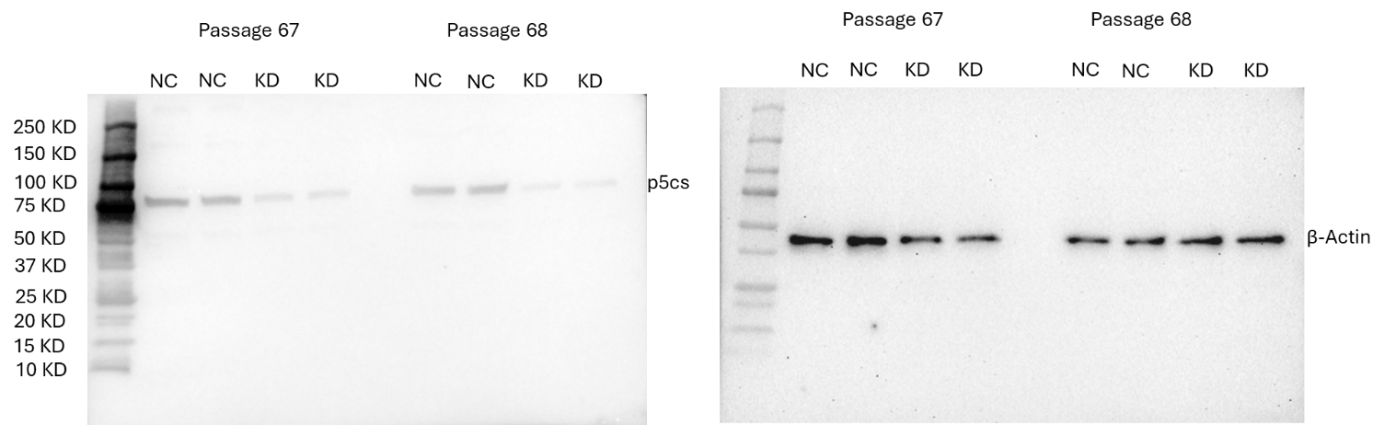


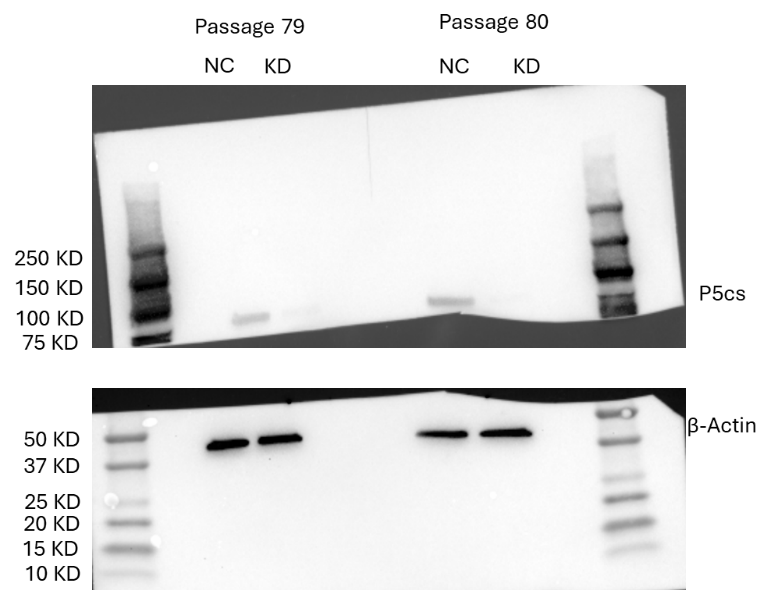


**Supplementary Fig. 2.** Uncropped blots showing P5CS protein expression following *ALDH18A1* knockdown in INS-1 832/13 cells across four different passages (n=4 independent experimental replicates).


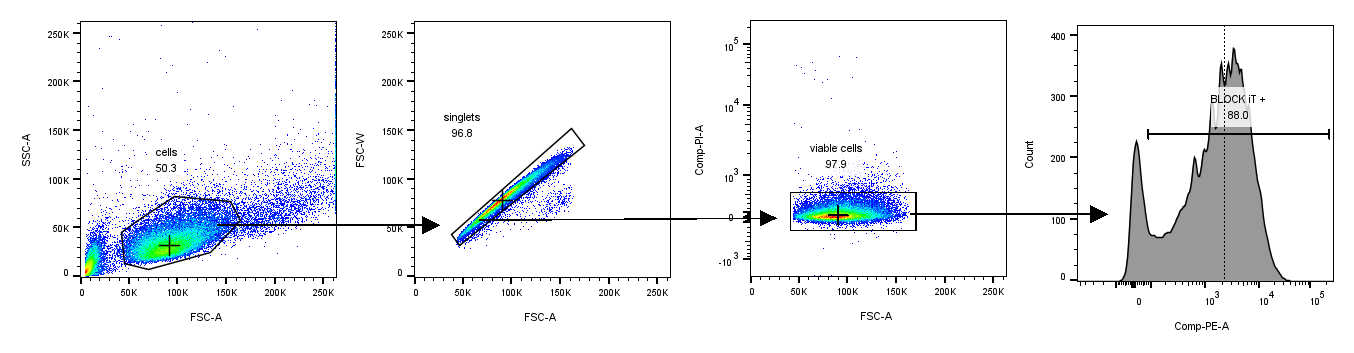


**Supplementary Fig. 3.** Evaluation of Transfection Efficiency in *ALDH18A1* Knockdown INS-1 832/13 Cells. The transfection efficiency was assessed using BLOCK-iT™ Alexa Fluor™ Red Fluorescent Control. The percentage of transfected cells was analyzed via flow cytometry (n=1 independent experimental replicate).
